# Select Formulæ for Diphtheria

**Published:** 1877-02-20

**Authors:** G. D. Hodge

**Affiliations:** Prescott, Ark.


### SELECT FORMULÆ FOR DIPHTHERIA.

By G. D. HODGE, M. D., Prescott, Ark

Diphtheria is already committing its fearful ravages in many portions of the "Sunny South," and, as the wails of weeping mothers are going up over the land, we hold that every true physician should be armed, and ready to fight, as best he can, this terrible scourge of the infantile race. With this view, we condense from past numbers of the RECORD a few formulæ, some one of which, in the hands of our brother practitioners, may, perchance, prove the means of restoring some little darling to the arms of its fond mother. If so, our sole aim will be gained.

R. Boiling water.................oz. 6—20
Liquid ses-guichlo. iron...m.20—60
Carbolic acid................ gr. 3
Red honey...................oz. 6 M.

One or two teaspoonsful every two hours internally, and as a gargle. *Keep up frictions of the skin from the beginning*, and use no cauterizations, active purgatives, or emetics. Prof. Lolli, of Italy, avers that this plan of treatment gives less than two per cent.

R. Chlo. pot. sat. sol........oz.4
Muriatic acid..............dr.1
Tinct. mur. iron..........dr. 2 M.

A small teaspoonful, properly diluted, every three hours, and alternate with 5 to 15 or 20 drops of liquor pot., for a child six years old.

R. Pulv. guaiac.

" myrrh.

" capsicum ...... *aa*..oz. 1

Alcohol...................oz. 16

From 5 to 30 drops in milk, alternated with 5 to 15 grs. of hyposulph. sod. every two to four hours. Over an hour should intervene between doses, to prevent coagulation of the milk. With chloral and glycerine locally, we have used both of the latter formulæ with signal benefit. Speaking of chloral, we believe, at no distant day, this agent, with bromide potassium, will "go to the front," as local remedies in throat affections.

R. Cincho-quinine ............gr. 3
Chlo. pot. sat. sol........dr.2
Salicylic acid...... .... ...gr.4
Tinct. mur. iron.........m.30
Brandy ... .................dr. 2
Sweet cream...............oz.2 M.

Use as an injection, every four to six hours. Increase or lessen the brandy, as necessity may require. If much jactitation exist, or should the enema be rejected, ten drops of laudanum may be added. So great are the absorptive powers of the rectum, that some extreme cases are reported as having been kept up for days, and finally conducted to convalescence with the above quieting, tonic and stimulating injection. We would think this plan well worthy of trial in cases in which we are debarred from medication otherwise.

